# Potential use of antibodies to provide an earlier indication of lymphatic filariasis resurgence in post–mass drug ad ministration surveillance in American Samoa

**DOI:** 10.1016/j.ijid.2022.02.006

**Published:** 2022-04

**Authors:** Angela M. Cadavid Restrepo, Katherine Gass, Kimberly Y. Won, Meru Sheel, Keri Robinson, Patricia M. Graves, Saipale Fuimaono, Colleen L Lau

**Affiliations:** a School of Public Health, Faculty of Medicine, The University of Queensland, Brisbane, Australia; b Research School of Population Health, College of Health and Medicine, Australian National University, Canberra, Australia; c Neglected Tropical Diseases Support Center, Task Force for Global Heath, Decatur, Georgia, United States of America; d Centers for Disease Control and Prevention, Division of Parasitic Diseases and Malaria, Atlanta, Georgia, United States of America; e National Centre for Epidemiology and Population Health, Research School of Population Health, College of Health and Medicine, Australian National University, Canberra, Australia; f College of Public Health, Medical and Veterinary Sciences, James Cook University, Cairns, Australia; g Department of Health, Pago Pago, American Samoa, United States of America

**Keywords:** Lymphatic filariasis, Post–mass drug administration, Antifilarial antibodies, Lymphatic filariasis resurgence, American Samoa, Transmission assessments surveys

## Abstract

**Background:**

Under the Global Programme to Eliminate Lymphatic Filariasis (LF), American Samoa conducted 7 rounds of mass drug administration (MDA) between 2000 and 2006. The territory passed transmission assessment surveys (TASs) in 2011 (TAS-1) and 2015 (TAS-2). In 2016, the territory failed TAS-3, indicating resurgence. This study aims to determine if antibodies (Abs) may have provided a timelier indication of LF resurgence in American Samoa.

**Methods:**

We examined school-level antigen (Ag) and Ab status (presence/absence of Ag- and Ab-positive children) and prevalence of single and combined Ab responses to Wb123, Bm14, and Bm33 Ags at each TAS. Pearson chi-square test and logistic regression were used to examine associations between school-level Ab prevalence in TAS-1 and TAS-2 and school-level Ag status in TAS-3.

**Results:**

Schools with higher prevalence of Wb123 Ab in TAS-2 had higher odds of being Ag-positive in TAS-3 (odds ratio [OR] 24.5, 95% confidence interval [CI] 1.2–512.7). Schools that were Ab-positive for WB123 plus Bm14, Bm33, or both Bm14 and Bm33 in TAS-2 had higher odds of being Ag-positive in TAS-3 (OR 16.0–24.5).

**Conclusion:**

Abs could provide earlier signals of resurgence and enable a timelier response. The promising role of Abs in surveillance after MDA and decision making should be further investigated in other settings.

## Highlights

• The patterns of the antibody (Ab) responses varied across the 3 transmission assessment surveys (TASs).
• The overall and school-level prevalence of Bm33 Ab was the highest in all TASs.
• Bm14 Ab-positive schools decreased, whereas Wb123 Ab-positive schools increased.
• Abs could provide earlier signals of resurgence and enable a timelier response.
• The role of Abs in post–mass drug administration surveillance should be further investigated.

## Introduction

Lymphatic filariasis (LF) is a parasitic infection caused by 3 species of the filarial nematodes, *Wuchereria bancrofti, Brugia malayi*, and *Brugia timori*, that are transmitted between definitive human hosts by multiple mosquito vectors (*Culex, Anopheles, Aedes*, and *Mansonia*) (Centers for Disease Control and Prevention). In 2000, the World Health Organization (WHO) targeted LF for global elimination by 2020 and launched the Global Programme to Eliminate Lymphatic Filariasis (GPELF) (WHO, 2000). One of the strategies proposed by GPELF focused on interrupting transmission by implementing mass drug administration (MDA) of antifilarial drugs in endemic areas (WHO, 2000). A key challenge faced by most LF-endemic countries that have implemented MDA is to effectively undertake postvalidation surveillance (Lau et al., 2020).

Transmission assessment surveys (TASs) are recommended by WHO in geographically defined evaluation units as the tool to measure the impact of MDA and determine whether the targets have been reached (WHO, 2011). School-based TASs are considered if attendance is high; otherwise, community cluster surveys are conducted. MDA is stopped when infection prevalence has been reduced to a level where it is presumed that transmission cannot be sustained even in the absence of further interventions. Estimates suggest that 4 to 6 annual rounds of MDA with effective population coverage (>65% of the total population) are required to reduce antigen (Ag) prevalence to <2% in areas where *Anopheles* or *Culex* is the main vector, and 1% where *Aedes* is the dominant vector (WHO, 2011). TAS is a population-based survey designed to estimate the prevalence of markers of LF infection in children aged 6 to 7 years. Because LF rapid Ag tests do not detect Brugia infections, TAS is done with rapid Ag tests in *W bancrofti*–endemic areas and rapid Ab tests in *Brugia*-endemic areas. This age group was selected because new incident infections would reflect recent exposure to ongoing transmission (WHO, 2011). According to WHO guidelines, TAS should also be repeated at 2 to 3 years and 4 to 6 years after stopping MDA in each evaluation unit to monitor and identify signals of resurgence (WHO, 2011).

In American Samoa, an MDA program to eliminate LF was initiated in 2000 under the Pacific Programme for the Elimination of Lymphatic Filariasis (WHO, 2006). Seven rounds of MDA with a single dose of diethylcarbamazine (DEC) and albendazole were conducted between 2000 and 2006 (WHO, 2006). In the first 3 years, population coverage by MDA was 24% to 52% and improved to 65% to 71% in the subsequent 4 years (WHO, 2006). American Samoa passed TAS-1 (in February 2011) and TAS-2 (in April 2015) with numbers of children who were Ag-positive below the critical cutoff of 6 (2 Ag-positive children in TAS-1 and 1 Ag-positive child in TAS-2, equivalent to crude prevalence and 95% confidence interval [CI] of 0.2% [0.0%–0.8%] and 0.1% [0.0%–0.7%], respectively) (Won et al., 2018a). However, the territory failed TAS-3 in November 2016, with 9 children who were Ag-positive with an adjusted prevalence of 0.7% (95% CI 0.3%–1.8%), which was higher than the cutoff and the recommended upper confidence limit of 1% (Sheel et al., 2018). In 2016, in parallel with TAS-3, a community survey of residents aged ≥8 years confirmed LF resurgence with adjusted Ag prevalence of 6.2% (95% CI 4.5%–8.6%). Spatial analyses of the 2016 community survey data also identified the potential existence of new or previously unidentified LF hotspots in the territory (Lau et al., 2020).

There is a current need to strengthen post-MDA surveillance through the development of alternative or additional surveillance strategies to identify residual LF infections and ensure long-term success of MDA. As LF elimination programs progress toward the end stages, one of the key challenges is the availability of diagnostics that are sufficiently sensitive for detecting low-level transmission or resurgence. Ag prevalence declines after the implementation of MDA, and as prevalence drops to low levels, more accurate tests and surveillance methods will be required to detect transmission signals. TAS currently relies on Ag test results only, and antigenemia alone may not be sensitive enough to ensure timely detection of ongoing transmission or recrudescence (Lau et al., 2020). Although the development and duration of serological responses (which indicate infection with the parasite) to specific antifilarial Abs such as Bm14, Bm33, and Wb123 are poorly understood, Ab testing may have a potential role in post-MDA and postvalidation surveillance in *W bancrofti*–endemic areas (Won et al., 2018b) because of the lack of a gold standard for infection detection by Ag tests.

Here, we examined the potential to use combinations of Ag and Ab tests as surveillance markers to provide earlier signals of transmission. This study aimed to geographically visualize and compare LF Ag and Ab signals in American Samoa at school level for TAS-1, TAS-2, and TAS-3 and to determine if antifilarial Abs in TAS-1 and TAS-2 may have provided an earlier indication of areas at risk for ongoing transmission in American Samoa.

## Methods

### Study setting

**Figure 1:**
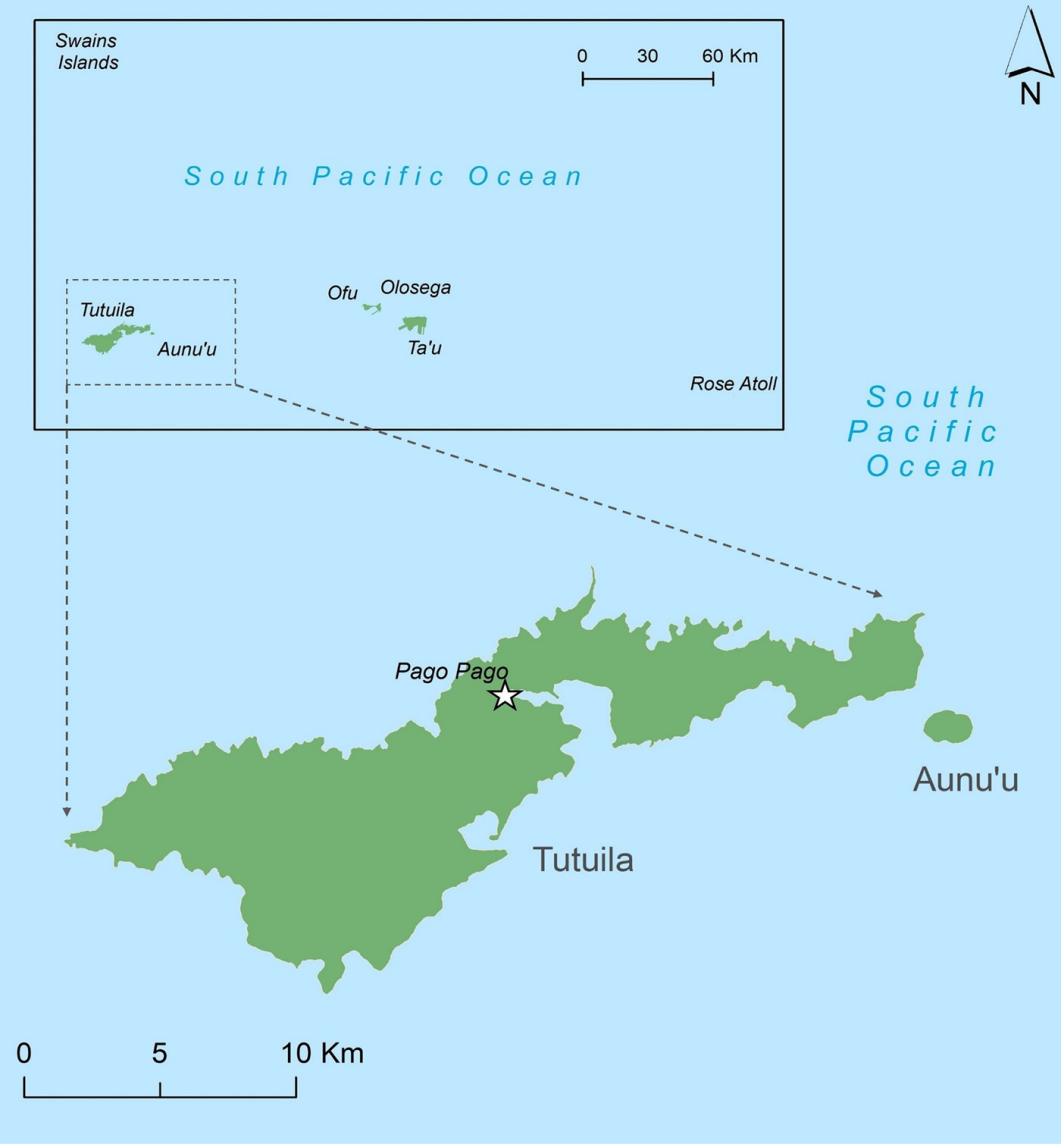
Map of American Samoa.

American Samoa is a US territory in the South Pacific and comprises 5 inhabited islands (Figure 1). The population was 55,519 in 2010, the majority of whom lived in the largest island, Tutuila (United States Census Bureau, 2010). The population is young, with one-thirds of the population younger than 15 years (United States Census Bureau, 2010). Education is compulsory between ages 6 and 18 and is provided by public and private elementary and secondary schools (Amerika Samoa Department of Education, 2020).

In American Samoa, LF is caused by *W bancrofti*, which are diurnally subperiodic worms transmitted predominantly by the highly efficient day-biting mosquito *Aedes polynesiensis* and also by the night-biting *Aedes samoanus* as a secondary vector (Schmaedick et al., 2014).

### Data sources

Data were obtained from 3 TASs conducted across American Samoa in 2011, 2015, and 2016. Surveys were carried out at 25, 30, and 29 schools for each survey year, respectively (all elementary schools on the main island of Tutuila and the adjacent island of Aunu'u). Because each TAS included children who attended grades 1 and 2 (used as a proxy for being 6–7 years old), each survey was conducted in a different cohort of children.

Informed written consent was obtained from a parent or guardian. Finger-prick blood samples (200 µL) were used to detect circulating filarial Ag. Binax NOW Filariasis Immunochromatographic Test (ICT) (Alere, Scarborough, ME) was used in TAS-1 and TAS-2, and Alere™ Filariasis Test Strip (FTS) (Abbott, Scarborough, ME) in TAS-3 (Abbott; Weil et al., 2013). Dried blood spots were prepared for later elution and antibody testing, where antifilarial Ab responses were tested by Luciferase Immunoprecipitation System (LIPS) assay (for IgG responses to Wb123 in TAS-1) or multiplex bead assay (MBA) (for antifilarial responses to Bm14 and Bm33 in TAS-1 and Wb123, Bm14, and Bm33 in TAS-2 and TAS-3) (Kubofcik et al., 2012; Lammie et al., 2012). A minimum of 4 controls were used for internal quality control for the MBA analyses. The first was a buffer blank that contained only the assay buffer, which was used to subtract any background noise. At least 2 controls were pools of reference sera that served as known positives for Abs to be detected in the assay. The last control was a negative control with known negative LF status. For TAS-1 and TAS-2, the cut-off determination methods have been previously described (Won et al., 2018a). For TAS-3 cut-off determination, the mean plus 3 standard deviations (SDs) method was used as it has been previously described (Moss et al., 2011; Priest et al., 2016).

Parents/guardians of children found to be Ag-positive were informed, and participants were offered a standard single dose of DEC (6 mg/kg) and albendazole (400 mg). Full details of survey designs and data collection have been reported elsewhere (Sheel et al., 2018; Won et al., 2018a).

An administrative boundary map was downloaded from the American Samoa Coastal and Marine Spatial Planning Data Portal (Marine Cadastre Admin, 2020). During TAS-3, the geographical coordinates of each school were collected using a hand-held global positioning system (Sheel et al., 2018) and imported into ArcGIS version 10.7.1 to create a shapefile of all elementary schools (ESRI: Environmental Systems Research Institute, 2019).

### Data analysis

A total of 33 schools were included in at least one of the surveys. Some elementary schools that participated in TAS-1, TAS-2, or in both were closed, and new schools were opened in the same/similar geographic area by the time TAS-3 was conducted. Thus, Vatia and Mt. Alava Elementary Schools located in the small village of Vatia, Olomoana, and Aoa schools located in Aoa village, and Iakina Adventist Academy and SDA located in Ili'ili village were considered as the same school in the analyses.

Because Ag and Ab prevalence at school level was low, the school Ag and Ab status was used for most analyses. Ag-positive schools were defined as those with at least 1 Ag-positive child. Ab-positive schools were defined as those with at least 1 child who tested positive to a single Ab or different combinations of Ab responses. For each TAS, summary statistics were calculated for the whole survey and for the 30 school locations. School-level crude prevalence of Ag, single Abs, and different combinations of Wb123, Bm14, and Bm33 Abs (see below) were estimated, and binomial exact methods were applied to estimate 95% CIs. Bar plots were created to show the prevalence of children who were Ab-positive in TAS-1 and TAS-2 stratified by the school Ag status (Ag-positive or Ag-negative) in TAS-3.

To enable comparisons over time, 5 schools that were not included in all 3 TASs were excluded from direct comparisons (Le'atele [Fagasa], Pacific Horizon, Peteli Academy, St. Theresa, and Ta'iala Academy). Therefore, only 25 school locations were included in the final analyses. The school Ab status in TAS-1 and TAS-2 and the school Ag status in TAS-3 were compared using Pearson chi-square tests. The following combinations of Ab responses at school-level in TAS-1 and TAS-2 were also examined to assess the value of testing a combination of Abs on diagnostic performance: (1) positive response to at least 1 Ab in the combinations, denoted henceforth as Wb123∪Bm14, Wb123∪Bm33, Bm14∪Bm33, and Wb123∪Bm14∪Bm33; and (2) positive response to all Abs in the combinations, denoted henceforth as Wb123∩Bm14, Wb123∩Bm33, Bm14∩Bm33, and Wb123∩Bm14∩Bm33. Sensitivity, specificity, positive predictive value (PPV), and negative predictive value (NPV) of Ab-positive schools in TAS-1 and TAS-2 for predicting Ag-positive schools in TAS-3 were also estimated.

Univariate logistic regression analyses were conducted to examine associations between single and combinations of Ab responses in TAS-1 and TAS-2 at school level and school Ag status in TAS-3. Haldane correction for odds ratio (OR) was used when either all children were Ag-positive or all were Ag-negative for a particular Ab response or combination of Ab responses (a value of 0.5 was added to every cell when cross‐product ratios of a 2 × 2 contingency table was zero) (Haldane, 1940; Lawson, 2004).

In all analyses, statistical significance was determined with α levels of 0.05 (as indicated by 95% CI). All analyses were conducted using R software version R-4.0.3 (R Core Team, 2020). Data were imported into ArcGIS version 10.7.1 (ESRI: Environmental Systems Research Institute, 2019) and linked spatially to the surveyed schools to generate maps that show the geographical distribution of the surveyed schools, the number of Ag-positive children identified through TAS, and crude Ab prevalence for each school.

## Results

**Figure 2:**
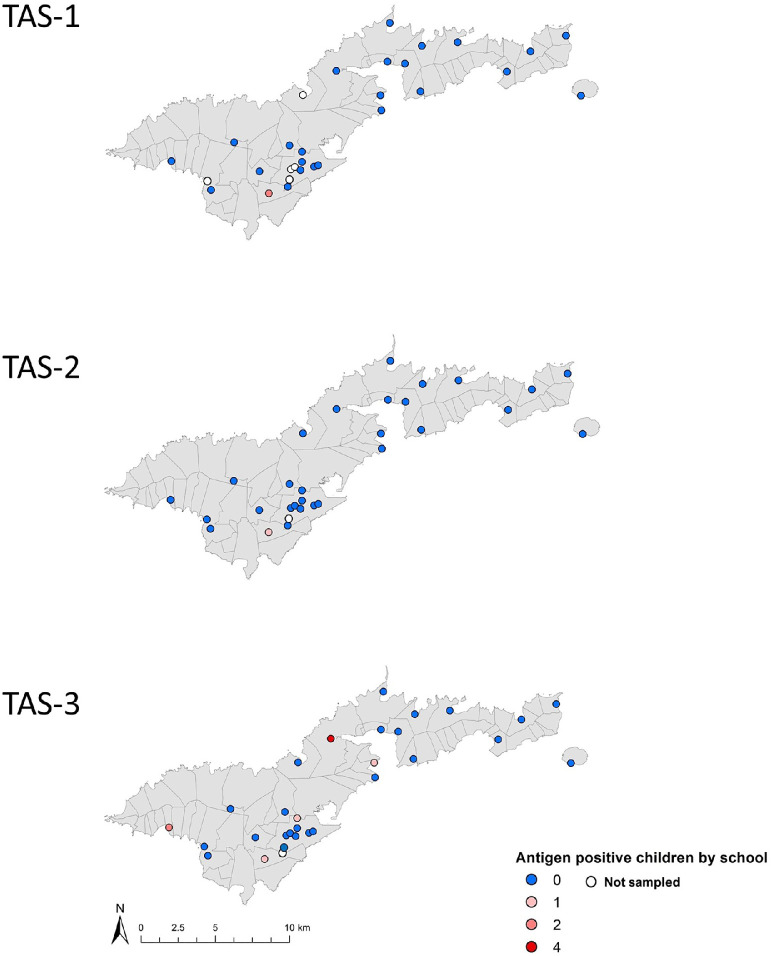
Locations of the schools (N = 30) included in the surveys and observed number of children who were antigen-positive in TAS-1 (2011), TAS-2 (2015), and TAS-3 (2016) in American Samoa. TAS, transmission assessment survey.

The initial data set consisted of 33 schools and a total of 1134 elementary school children who participated in TAS-1, 864 in TAS-2, and 1143 in TAS-3. The overall crude Ag prevalence was 0.2% (95% CI 0%–0.8%) in TAS-1 (n = 937), 0.1% (95% CI 0%–0.7%) in TAS-2 (n = 768), and 0.8% (95% CI 0.4%–1.5%) in TAS-3 (n = 1143) (Table 1 and Supplementary Figure). After adjusting for survey design and age and sex distribution, Ag prevalence was 0.7% (95% CI 0.3%–1.8%) in TAS-3 (Sheel et al., 2018). Lupelele Elementary was the only school with Ag-positive school children in TAS-1 (2 of 92 children tested positive, Ag prevalence 2.2%, 95% CI 0.26%–7.63%) and TAS-2 (1 of 85 children tested positive, Ag prevalence 1.2%, 95% CI 0%–6.4%). In addition, Lupelele Elementary was among the 5 schools with children who were Ag-positive in TAS-3. In TAS-3, Ag prevalence by school ranged from 0% to 4.9%; Coleman Elementary had the highest number of students who were Ag-positive (Supplementary Table 1). School locations included in the 3 surveys and the number of children who were Ag-positive identified through TAS are shown in Figure 2.

**Table 1:** Summary of participants and results from TAS-1 (2011), TAS-2 (2015), and TAS-3 (2016) surveys in American Samoa.

	TAS-1	TAS-2	TAS-3
Timing	February 2011	April 2015	September 2016
Total number of schools	25	30	29
Total number of participants	1134	864	1143
Number of participants with valid Ag test results	937	768	1143
Number of children who were Ag-positive	2	1	9
Crude Ag prevalence (95% CI)	0.2% (0.0–0.8)	0.1% (0.0–0.7)	0.7% (0.4–1.5)
Number of participants with Ab test results	1112	836	1139
Number of children who were Ab-positive and crude prevalence (%) Wb123 Bm14 Bm33Number and % of schools with at least 1 child who was Ab-positive Wb123 Bm14 Bm33Number and % of schools that were positive for at least 1 Ab in the following combinations Wb123∪Bm14 Wb123∪Bm33 Bm14∪Bm33 Wb123∪Bm14∪Bm33Number and % of schools that were positive for all Abs in the following combinations Wb123∩Bm14 Wb123∩Bm33 Bm14∩Bm33 Wb123∩Bm14∩Bm33	11(1.0%)76 (6.8%)133 (12.0%)6 (24.0%)22 (88.0%)20 (80.0%)22 (88.0%)20 (80.0%)25 (100.0%)25 (100.0%)6 (24.0%)6 (24.0%)17 (68.0%)6 (24.0%)	30 (3.6%)25 (3.0%)65 (7.8%)12 (40.0%)12 (40.0%)17 (56.7%) 14 (46.6%)17 (56.6%)18 (60.0%)18 (60.0%)8 (26.7%)11 (36.7%)10 (33.3%)8 (26.7%)	94 (8.3%)18 (1.6%)237 (20.8%)22 (75.9%)10 (34.5%)25 (86.2%)21 (72.4%)24 (82.8%)22 (75.9%)24 (82.6%)9 (31.0%)20 (67.0%)10 (34.5%)9 (31.0%)

Ab, antibody; Ag, antigen; CI, confidence interval; TAS, transmission assessment survey.

**Figure 3:**
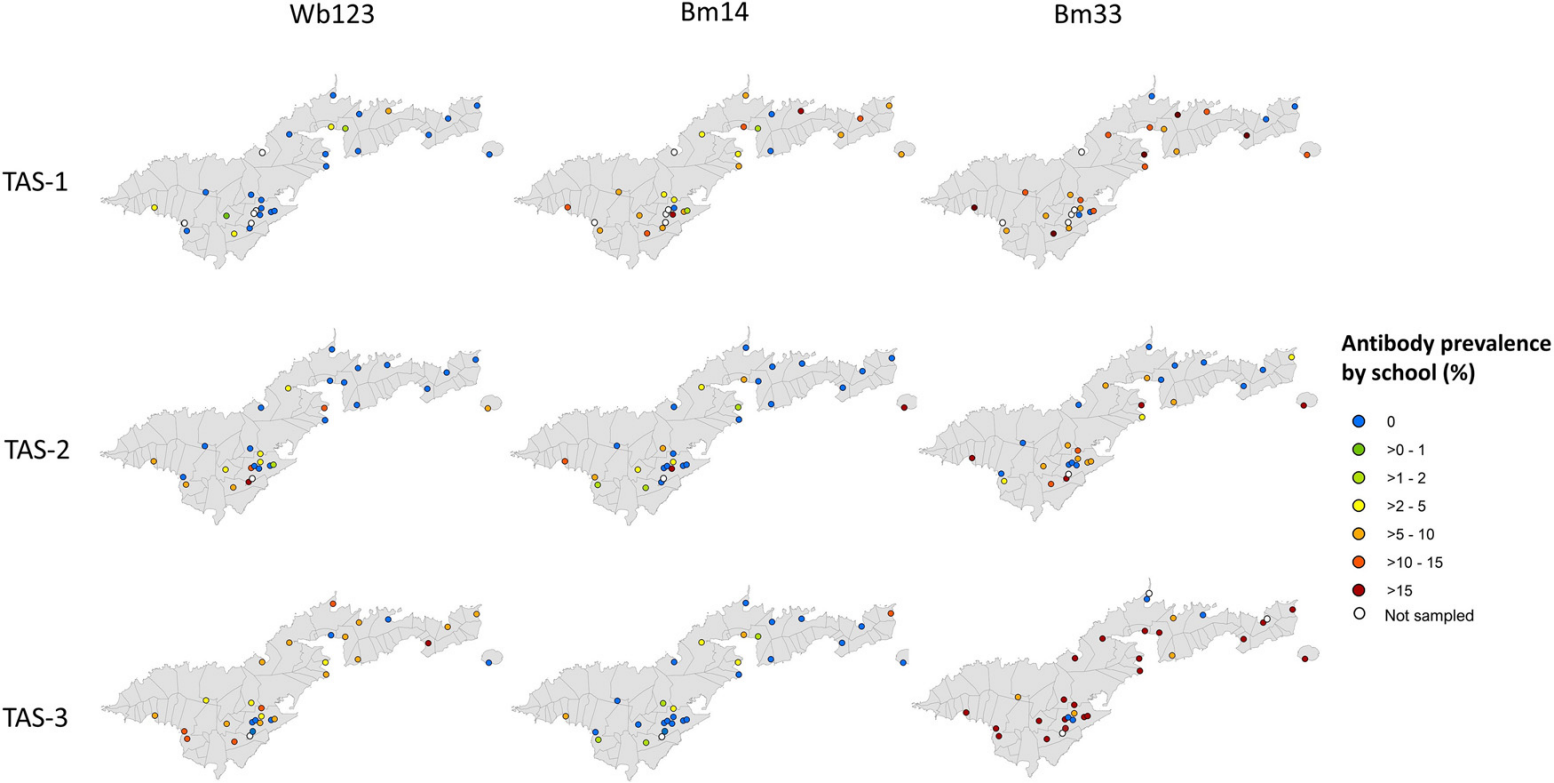
School locations (N = 30) included in surveys and prevalence of antibody responses to Wb123, Bm14, and Bm33 in TAS-1 (2011), TAS-2 (2015), and TAS-3 (2016) in American Samoa. TAS, transmission assessment survey.

**Figure 4:**
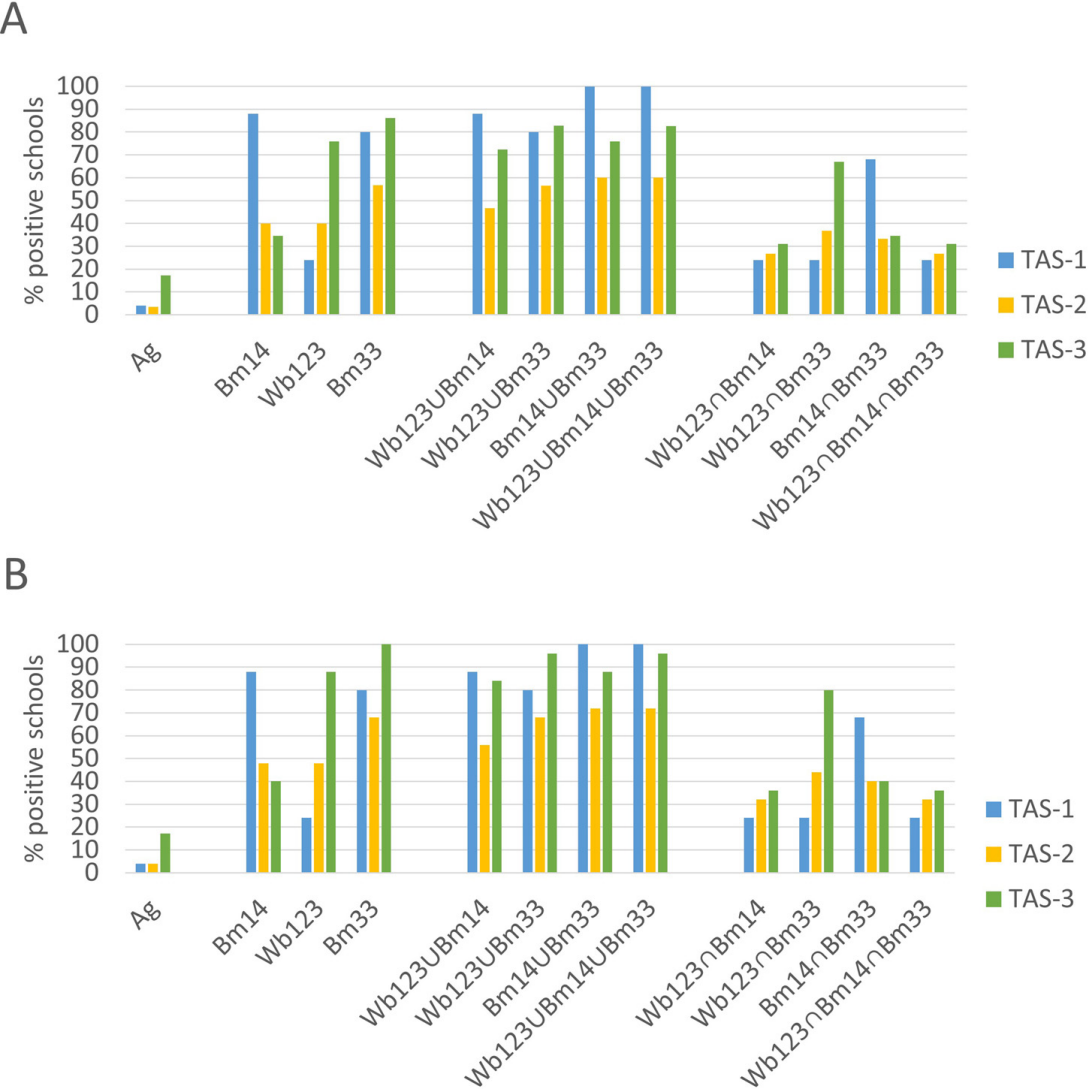
Percentage of Ag- and Ab-positive schools (including all combinations of Abs) among (A) all school locations (n = 30) that participated in TAS-1, TAS-2, and TAS-3, and (B) the 25 schools that participated in all 3 TAS. Ab, antibody; Ag, antigen; TAS, transmission assessment survey.

In TAS-1, results of Ab responses were available for 1112 schoolchildren; the highest overall Ab prevalence was observed for Bm33 (12.0%, 95% CI 10.1%–14.0%), followed by Bm14 (6.8%, 95% CI 5.4%–8.5%), and Wb123 (1.0%, 95% CI 0.5%–17.6%). In TAS-2 and TAS-3, Ab results were available for 836 and 1139 children, respectively; responses to Bm33 were also the highest in both surveys (7.8%, 95% CI 6.1%–9.8% in TAS-2 and 20.8%, 95% CI 18.5%–23.3% in TAS-3) (Supplementary Table 2) Figure 3. shows the school-level Ab prevalence for Wb123, Bm14, and Bm33 in each survey. All children who were Ag-positive in TAS-1 and TAS-2 were seropositive for all 3 Abs. Of the 9 children who were Ag-positive in TAS-3, 6 were seropositive for all Abs, and the remainder were seropositive for at least 1 Ab (Supplementary Table 3). Considering Ag and Ab status at school level (presence or absence of Ag- and Ab-positive children), the percentage of Bm14 Ab-positive schools decreased, whereas the percentage of Wb123 Ab-positive schools increased over time (Figure 4).

**Figure 5:**
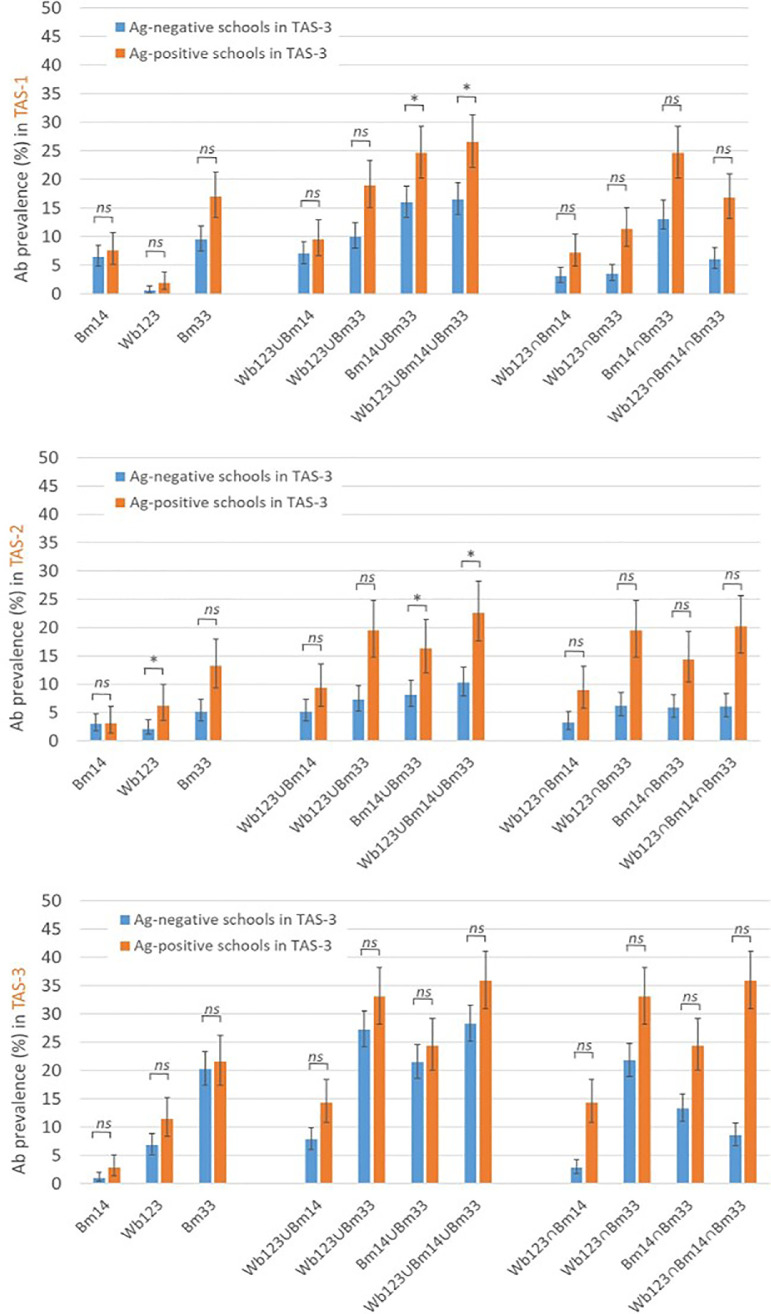
Crude prevalence of Ab-positive responses to single (Bm14, Bm33, Wb123) and combinations of Ab responses (Wb123∪Bm14, Wb123∪Bm33, Bm14∪Bm33, Wb123∪Bm14∪Bm33, Wb123∩Bm14, Wb123∩Bm33, Bm14∩Bm33, and Wb123∩Bm14∩Bm33) in TAS-1, TAS-2, and TAS-3 among children from the 25 schools that participated in all 3 TASs, stratified by school Ag status (presence or absence of children who were Ag-positive) in TAS-3. *p ≤ 0.05; ns indicates *p* > 0.05. Ab, antibody; Ag, antigen; ns, not significant; TAS, transmission assessment survey.

The percentage of children who were Ab-positive stratified by the school Ag status at TAS-3 was also examined. In each TAS, Ab prevalence was higher in children who attended schools that were Ag-positive in TAS-3 than those who attended Ag-negative schools in TAS-3. In general, single Ab results in TAS-1 and Ag-negative schools in TAS-2 follow a similar trend with the highest prevalence for Bm33 Ab, followed by Bm14 and Wb123. A shift was observed in TAS-2 for schools that were Ag-positive in TAS-3 and all schools in TAS-3 toward higher prevalence of Wb123 than Bm14 Ab (Figure 5).

### Associations between school Ab status in TAS-1 and TAS-2 and school Ag status in TAS-3

To assess whether the school's Ab-positive status at 1 survey time point was associated with Ag-positive status in a later survey, we compared these seromarkers in TAS-3 and previous surveys. At the school level, significant statistical association was found between Wb123 Ab-positive status in TAS-2 and Ag-positive status in TAS-3 (χ^2^_1_ = 5.36, *p* = 0.02). Chi-square tests also show that some combinations of positive Abs (Bm14∪Bm33 and Wb123∪Bm14∪Bm33) in TAS-1 and TAS-2 were also statistically significantly associated with Ag-positive school status in TAS-3 (both with the same χ^2^_1_ = 9.00, *p* = 0.002). Positive responses to Bm33, Bm14, Wb123∪Bm14, Wb123∪Bm33, Bm14∪Bm33, Wb123∪Bm14∪Bm33, and Bm14∩Bm33 in TAS-1, and all single and combinations of Ab responses in TAS-2 would have predicted Ag-positive schools in TAS-3 with high sensitivity (>80%) but low to moderate specificity (25%–80%). From the positive Ab responses in TAS-2, Wb123∩Bm14 and Wb123∩Bm14∩Bm33 had the highest sensitivity (80%, 95% CI 29%–99%) and specificity (80%, 95% CI 56%–94%) results. The findings also revealed that Wb123 alone, Wb123∩Bm14, Wb123∩Bm33, and Wb123∩Bm14∩Bm33 in TAS-1 were less sensitive (40%, 95% CI 5%–85%) indicators of children who were Ag-positive in TAS-3 but were more specific (80%, 95% CI 56%–94%) (Table 2).

**Table 2:** Sensitivity, specificity, PPV, and NPV for using school Ab status in TAS-1 and TAS-2 as indicators of school Ag status in TAS-3.

Indicator	School Ab status	Number of total Ab + schools in TAS-1 or TAS-2	Number of schools Ab+ in TAS-1 or TAS-2 and Ag-positive in TAS-3	Sensitivity % (95% CI)	Specificity % (95% CI)	PPV % (95% CI)	NPV % (95% CI)
Ab responses in TAS-1 as indicator of Ag-positive schools in TAS-3	Positive for single Ab						
	Wb123	6	2	40 (5–85)	80 (56–94)	33 (4–78)	84 (60–97)
	Bm14	22	5	100 (48–100)	15 (5–38)	23 (8–45)	100 (29–100)
	Bm33	20	5	100 (48–100)	25 (9–49)	25 (9–49)	100 (48–100)
	Positive for at least 1 Ab in the combination						
	Wb123∪Bm14	22	5	100 (48–100)	15 (3–38)	23 (8–45)	100 (29–100)
	Wb123∪Bm33	20	5	100 (48–100)	25 (9–49)	25 (9–49)	100 (48–100)
	Bm14∪Bm33	25	5	100	0	20	0
	Wb123∪Bm14∪Bm33	25	5	100	0	20	0
	Positive for all Abs in the combination						
	Wb123∩Bm14	6	2	40 (5–85)	80 (56–94)	33 (4–78)	84 (60–97)
	Wb123∩Bm33	6	2	40 (5–85)	80 (56–94)	33 (4–78)	84 (60–97)
	Bm14∩Bm33	17	5	100 (48–100)	40 (19–64)	29 (10–56)	100 (63–100)
	Wb123∩Bm14∩Bm33	6	2	40 (5–85)	80 (56–94)	33 (4–78)	84 (60–97)
Ab responses in TAS-2 as indicators of Ag-positive schools in TAS-3	Positive for single Ab						
	Wb123	11	5	100 (48–100)	70 (46–88)	45 (17–77)	100 (77–100)
	Bm14	11	4	80 (28–99)	65 (41–85)	36 (11–69)	93 (66–100)
	Bm33	17	5	100 (48–100)	40 (19–64)	29 (10–56)	100 (63–100)
	Positive for at least 1 Ab in the combination						
	Wb123∪Bm14	14	5	100 (48–100)	55 (32–77)	36 (13–65)	100 (72–100)
	Wb123∪Bm33	17	5	100 (48–100)	40 (19–64)	29 (10–56)	100 (63–100)
	Bm14∪Bm33	18	5	100 (48–100)	35 (15–59)	28 (10–53)	100 (59–100)
	Wb123∪Bm14∪Bm33	18	5	100 (48–100)	35 (15–59)	28 (10–53)	100 (59–100)
	Positive for all Abs in the combination						
	Wb123∩Bm14	8	4	80 (28–99)	80 (56–94)	50 (16–84)	94 (71–100)
	Wb123∩Bm33	11	5	100 (48–100)	70 (46–88)	45 (17–77)	100 (77–100)
	Bm14∩Bm33	10	4	80 (28–99)	70 (46–88)	40 (12–74)	93 (68–100)
	Wb123∩Bm14∩Bm33	8	4	80 (28–99)	80 (56–94)	50 (16–84)	94 (71–100)

Gray: 0%–25%; light blue: 26%–50%; medium blue: 51%–79%; dark blue: 80%–100%.

Ab, antibody; Ag, antigen; CI, confidence interval; NPV, negative predictive value; PPV, positive predictive value; TAS, transmission assessment survey.

### Prediction of school Ag status in TAS-3 based on school Ab status in TAS-1 and TAS-2

The results of the regression analyses (Table 3) indicate that Wb123 Ab-positive schools in TAS-2 were significantly associated with Ag-positive status in TAS-3 (OR 24.5, 95% CI 1.17–512.6). Similarly, schools that were positive for Wb123∩Bm14, Wb123∩Bm33, and Wb123∩Bm14∩Bm33 in TAS-2 also had higher odds of being Ag-positive in TAS-3 (OR ranging from 16.0 to 24.5). Schools that were Ab-positive for Bm14∪Bm33 and Wb123∪Bm14∪Bm33 in TAS-1 also had significantly higher odds of being Ag-positive in TAS-3.

**Table 3:** Antibody positivity (at school level) in TAS-1 and TAS-2 as predictors of school Ag status in TAS-3.

Predictors		Number of Ag-positive schools in TAS-3	OR	95% CI
TAS-1	**Positive for individual Ab**			
	Wb123	2	2.6	0.3–21.7
	Bm14	5	2.2^a^	0.1–49.5
	Bm33	5	3.9^a^	0.2–82.8
	**Positive for at least 1 Ab in the combination**			
	Wb123∪Bm14	5	2.2^a^	0.1–49.5
	Wb123∪Bm33	5	3.9^a^	0.2–82.8
	Bm14∪Bm33	5	451^a^	0.8–25,409
	Wb123∪Bm14∪Bm33	5	451^a^	0.8–25,409
	**Positive for all Abs in the combination**			
	Wb123∩Bm14	5	2.6	0.3–21.7
	Wb123∩Bm33	5	2.6	0.3–21.7
	Bm14∩Bm33	5	7.4^a^	0.4–153.8
	Wb123∩Bm14∩Bm33	5	2.6	0.3–21.7
**TAS-2**	**Positive for individual Ab**			
	Wb123 Ab	5	**24.5**^a^	**1.2**–**512.7**
	Bm14 Ab	4	7.4	0.7–80.0
	Bm33 Ab	5	7.5^a^	0.4–143.8
	**Positive for at least 1 Ab in the combination**			
	Wb123∪Bm14	5	13.3^a^	0.7–272.8
	Wb123∪Bm33	5	7.5^a^	0.4–153.8
	Bm14∪Bm33	5	2.6^a^	0.3–21.7
	Wb123∪Bm14∪Bm33	5	2.6^a^	0.3–21.7
	**Positive for all Abs in the combination**			
	Wb123∩Bm14	5	**16.0**	**1.4**–**185.4**
	Wb123∩Bm33	5	**24.5**^a^	**1.2**–**512.6**
	Bm14∩Bm33	5	2.7	0.3–21.7
	Wb123∩Bm14∩Bm33	5	**16.0**	**1.4**–**185.4**

a Note that accurate ORs could not be calculated because all Ab-positive schools in these categories were Ag-positive in TAS-3. Reported ORs were calculated after applying Haldane correction. Ab, antibody; Ag, antigen; CI, confidence interval; OR, odds ratio; TAS, transmission assessment survey.

## Discussion

Our study compared Ag and Ab results obtained in the 3 TASs conducted in American Samoa in 2011, 2015, and 2016. We found that the school Ab statuses in TAS-1 and TAS-2 were significantly associated with the presence of Ag-positive children in TAS-3 and could have provided an earlier indication of resurgence than the use of Ag alone. The results suggest that antifilarial Ab responses among young children may be used as early signals of ongoing transmission or resurgence in a post-MDA setting. The findings also showed that the statistically significant associations between responses to Wb123, Wb123∩Bm14, and Wb123∩Bm14∩Bm33 at school level in TAS-2 and Ag-positive schools in TAS-3 provided the best balance of sensitivity (80%) and specificity (80%) test results.

The serological patterns of the Ab responses to Wb123, Bm14, and Bm33 Ags varied across the 3 TASs. The overall and school-level prevalence of Bm33 Ab was the highest in all TASs. In addition, Bm33 Ab and all sets of combinations that included this Ab were highly prevalent in both Ag-positive and Ag-negative schools in TAS-3. These findings concur with previous studies that found Bm33 Ab as the first detectable seromarker that induces an Ab response even without high levels of antigenemia (Hamlin et al., 2012). In contrast, the prevalence of Wb123 Ab increased from TAS-1 to TAS-3. The kinetics of Wb123Ab are not currently well understood. It has been proposed that the response to Wb123 is developed after repeated larval stimulation, which is then required to sustain an Ab response to Wb123 (Kubofcik et al., 2012). Therefore, it is expected that as infection rates increase, the prevalence of Wb123 responses will follow the same trend. The decrease in prevalence of Bm14 Ab over the 3 TASs was an unexpected serological pattern that differed from previous reports (Hamlin et al., 2012). The discordant results between Ag and the 3 Abs suggest that serological responses in recently acquired infections are complex and that further studies are required to fully understand and interpret Ag and Ab profiles (Moss et al., 2011). In pre-MDA settings, it is expected that Ab responses are concordant as there is no variability in the intensity of Ag exposure (Hamlin et al., 2012). After MDA interventions, studies conducted in different settings have shown that Ab responses tend to decline (Ramzy et al., 2006; Tisch et al., 2008). In older age groups, the persistence of Ag and Ab for long periods makes their use challenging for identifying ongoing transmission (Lau et al., 2020). However, existing data indicate that antibody responses are not lifelong and can inform LF status at a population level (Helmy et al., 2006; Weil et al., 2008; Won et al., 2018b).

Increasing evidence indicates that the use of Ag alone in TASs may not be sufficiently sensitive for making decisions to stop MDA or for post-MDA surveillance (Gass et al., 2012; Hamlin et al., 2012; Won et al., 2018b). Although Sri Lanka was recognized as having eliminated LF as a public health problem in 2016, more recent studies found low-level persistence of infection in some regions (Rao et al., 2018). Similarly, other LF-endemic areas including Tonga, American Samoa, and India, which had used school-based or community TASs for post-MDA surveillance also found that TASs alone were not sufficiently sensitive for programmatic decision making (Joseph et al., 2011; Lau et al., 2020; Subramanian et al., 2020).

In the context of American Samoa, although the territory passed TAS-1 and TAS-2, the surveys failed to detect hotspots and residual ongoing transmission, resulting in a resurgence of LF and ultimately failing TAS-3 (Sheel et al., 2018). Therefore, alternative surveillance methods are required to improve the prompt identification of ongoing transmission or resurgence in low-prevalence settings. This is particularly important in the post-MDA setting when residual infections can be highly spatially heterogeneous (Lau et al., 2014).

As initiatives to incorporate Ab testing as a tool to strengthen post-MDA surveillance are increasingly been proposed, additional work is needed to assess the performance of Ab assays and develop more species-specific tests to complement TAS. This is particularly important in areas of coendemicity with other filarial infections, where cross-reactivity with Ags from other filarial parasites such as *Onchocerca volvulus* and *Loa* have been documented (Dolo et al., 2019; Hertz et al., 2018).

The limitations of this study include the use of different Ag tests and Ab assays over the 3 TASs. The use of different cut-off values to define Ag- or Ab positivity also pose challenges for the interpretation of results. Therefore, further work is needed to standardize tests and revise and propose consistent cut-off thresholds for MBA. A recent study assessed the concordance between ICT and FTS results using blood samples collected in 2016 in American Samoa (Sheel et al., 2021). The study found that the difference in Ag prevalence between the 2 Ag tests was not statistically significant and that the results of TAS-3 would have been very similar with either Ag test. At the time of TAS-1, LIPS was the only option available for measuring Wb123 Ab, and samples from TAS-1 and TAS-2 were not repeated when TAS-3 was completed. Unfortunately, it is not always feasible to repeat the testing of samples from large studies or to perform concurrent testing from multiple surveys conducted at different times. Therefore, the inability to retest samples and to use a consistent platform may be a limitation for the comparison of Wb123 Ab results over time.

This study provides important evidence that helps better understand antifilarial Abs in children in the context of LF resurgence. The associations found between the school Ab status in TAS-1 and TAS-2 and the school Ag status in TAS-3 suggest that Abs could have provided an earlier indication of LF resurgence in American Samoa. Although our study was conducted on data from American Samoa, the concepts are widely applicable to other settings globally. Our findings contribute new evidence for the potential role of Ab testing as an additional monitoring tool that may help guide programmatic decision making and strengthen post-MDA surveillance. Further studies are required to better understand specific Ab responses after MDA.

## Author contributions

AMCR, CLL, and PMG developed the study concept and design. Analyses were performed by AMCR and CLL. AMCR and CLL drafted the manuscript. All authors helped in the interpretation of results and critically reviewed the manuscript.

## Data sharing

The data used in the present study are available from the corresponding author on reasonable request.

## Ethical approvals

Ethics approvals for TAS-1 and TAS-2 were granted by the American Samoa Department of Health Institutional Review Board and the U.S. Centers for Disease Control and Prevention as program evaluation, nonresearch (Won et al., 2018a). TAS-3 was approved by the American Samoa Institutional Review Board and the Human Research Ethics Committee at the Australian National University (protocol number 2016/482) and the University of Queensland (2021/HE000896). Full details of collaborations and official permissions for school and village visits in 2016 have been previously described (Sheel et al., 2018).

## Funding

CLL was supported by Australian National Health and Medical Research Council Fellowships (APP1193826). MS is supported by a fellowship from the Westpac Scholars Trust.

## Declaration of interests

The authors declare no competing interests.

## Appendix Supplementary materials

Image, application 1
